# Implementing mental health training programmes for non-mental health trained professionals: A qualitative synthesis

**DOI:** 10.1371/journal.pone.0199746

**Published:** 2018-06-25

**Authors:** Arabella Scantlebury, Adwoa Parker, Alison Booth, Catriona McDaid, Natasha Mitchell

**Affiliations:** 1 Institute of Health and Society, Newcastle University, Newcastle Upon-Tyne, England; 2 York Trials Unit, Department of Health Sciences, University of York, York, England; Northumbria University, UNITED KINGDOM

## Abstract

**Introduction:**

Given the prevalence of mental health problems globally, there is an increasing need for the police and other non-mental health trained professionals to identify and manage situations involving individuals with mental health problems. The review aimed to identify and explore qualitative evidence on views and experiences of non-mental health professionals receiving mental health training and the barriers and facilitators to training delivery and implementation.

**Methods:**

A meta-synthesis of qualitative evidence on the barriers, facilitators and perceived impact of mental health training programmes for non-mental health trained professionals. Systematic literature searches were undertaken of the following databases: Criminal Justice Abstracts (CJA); MEDLINE; Embase; PsycINFO; ASSIA; CENTRAL; SSCI; ERIC; Campbell Library; Social Care Online and EPOC from 1995 to 2016. Records were independently screened for eligibility by two researchers, data extraction and quality appraisal of studies was also undertaken independently by two researchers. The CASP tool was used to quality appraise included studies. Included studies were synthesised using a meta-ethnographic approach as outlined by Noblit and Hare.

**Results:**

10,282 records were identified and eight qualitative studies were included. A range of barriers and facilitators to training were identified and related to the delivery and content of training; the use of additional resources; and staff willingness to engage with training and organisational factors. The perceived impact of training was also discussed in terms of how it affects trainees; perceptions of mental health; self-perception; responses to situations involving mental health and the potential of training to reduce injury or physical harm in situations involving mental health. The value of training and how to measure its impact were also discussed.

**Conclusion:**

Findings from this review have implications for those designing, implementing and evaluating mental health training programmes. It is recommended that research evaluating mental health training includes a qualitative component to ensure that the barriers and facilitators to training and its impact on trainees’ perceptions of mental health are understood.

**Protocol registration number:**

PROSPERO: CRD42015015981

## Introduction

Mental health problems are one of the main causes of disease burden worldwide, with five types of mental illness appearing in the top 20 causes of global burden of disease: major depression, anxiety disorders, schizophrenia, dysthymia and bi-polar disorder [1]. In the UK, the current climate of austerity and cuts to mental health services have contributed to concern that police officers are being relied on as a first resort to incidents involving individuals with mental health problems [2]. In 2015, the UK College of Policing reported increased levels of demand in responding to people with mental health problems, with an estimated 15–20% of police time spent on incidents linked to mental health in England and Wales [3]. Police officers are not expected to be experts in mental health, or deal with this vulnerable group in isolation. However, police officers are often the first to respond to situations involving individuals experiencing mental crisis [4] and so are expected to be able to recognise the ‘warning signs’ and work with health and social care agencies; to ensure that an appropriate response is provided [5, 6].

The need for police officers to receive mental health training has been recognised. In the US over 400 Crisis Intervention Teams (CIT) have been introduced which aim to enhance how police officers interact with, and respond to situations involving mental health crisis, through the provision of mental health training [7]. This has also been recognised in other countries such as the UK, where the National Policing Improving Agency has emphasised the need for mental health training for police officers [6]. However, the extent of training provided to police officers varies and it is unclear what the most effective approaches are to training police officers in the identification and management of mental health.

The current review is part of a broader systematic review of mental health training for non-mental health trained professionals (PROSPERO record CRD42015015981)[8]. The purpose of the systematic review was to inform the development of a training programme for police officers, that was evaluated by a Randomised Controlled Trial (RCT) (ISRCTN registry trial ID ISRCTN11685602). Our main interest at the outset was in training for police officers. However, our preliminary searches and discussions with people working in the field suggested there may be limited studies available on mental health training for police officers. As a result, the scope of the review was widened to include other non-mental health trained occupational groups who, as part of their work, come into contact with people with mental health problems (e.g. teachers).By widening the scope of our review, we hoped to capture a broader range of perceptions and experiences of mental health training that would be transferable to the police setting.

The systematic review was designed to (i) collate the quantitative evidence on the effectiveness of mental health training interventions for non-mental health qualified professionals and (ii) collate the qualitative evidence on the views and experiences of non-mental health professionals receiving mental health training and barriers and facilitators to training delivery and implementation. Given, the volume and richness of the qualitative data identified the review of quantitative studies of effectiveness are reported separately[8]. This meta-synthesis aims to complement the systematic review of quantitative evidence on the effectiveness of training programmes by identifying and exploring qualitative evidence on the views and experiences of training and barriers and facilitators to its delivery and implementation. Findings from the review informed the development of a bespoke mental health training program for police officers that was evaluated using a RCT.

## Methods

### Searching and identifying relevant studies

An information specialist undertook the searches. Search strategies (S1 Text) were adapted and implemented in the following databases: Criminal Justice Abstracts (CJA); MEDLINE; Embase; PsycINFO; ASSIA; CENTRAL; SSCI; ERIC; Campbell Library; Social Care Online and EPOC. Manual searches of the reference lists of included studies were also undertaken. The websites of major mental health charities (MIND, Rethink, Black Mental Health UK and YoungMinds) were searched and contacted for relevant studies and evaluations of training.

### Inclusion and exclusion criteria

The inclusion and exclusion criteria for the review of qualitative studies are reported in Table 1. For the purposes of this meta-synthesis non-mental health trained professionals are any individuals that have not received mental health training, other than anything that they may have received as part of their professional basic training and are working in the criminal justice system, education, health service or any other organisation who interact with the public (Table 1). The introduction of the Mental Health Act (1983)[9] led to the production of a number of seminal reports which aimed to address public attitudes to mental health and a co-ordinated response from the police in responding to incidents involving mental health [6, 10, 11]. In light of changing legislation, attitudes and awareness of mental health in the UK, evidence from the last 20 years was included. An English language only restriction was used, with papers from OECD countries included.

**Table 1:** Inclusion and exclusion criteria adapted from SPIDER [12].

Inclusion	Exclusion
**Sample:** Police officers; staff employed by the Police who come into contact with the public (e.g. Force Control room staff), members of other parts of the criminal justice system (e.g. prison officers), non-mental health trained health professionals (e.g. paramedics), people working in education, any other professions or organisations who interact with the public in a similar way to the police, people working for relevant charities (e.g. MIND, YoungMinds).	Mental health trained professionals
**Phenomenon of interest:** Mental health training	Mental health awareness training delivered as part of a basic training package to newly appointed Police staff
**Design:** Interviews, focus groups, open-ended surveys and observational studies. Audits and evaluations of mental health training for mental health charities and English and Welsh Police forces.	
**Evaluation:** Courses, training, learning packages or other resources that sought to increase knowledge of mental health and/or changing attitudes and/or improving their skills in dealing with mental health problems.	Training which did not primarily aim to improve knowledge or change behaviour and/or attitudes towards mental health. For example, training that sought to improve how individuals interact with an elderly population, which may include dementia training was not included.
**Research Type:** Qualitative and mixed methods studies	

### Data extraction

Titles and abstracts and then potentially relevant full papers were independently screened by two reviewers, discrepancies were resolved through discussion. No third party resolution was required. A data extraction form was piloted and information relating to: country, setting, participants, study aims, training intervention, method of evaluation and methodology, views and experiences of training and barriers and facilitators to implementation were independently extracted by one reviewer and checked by another.

### Literature synthesis

Although debate exists as to whether meta-ethnography can be applied to non-ethnographic studies, the method has been applied to a number of meta-syntheses, [13–16]; possibly as a result of the guidance provided by Noblit and Hare [17]. We adapted Noblit and Hare’s guidance, with the analysis of qualitative studies comprising six iterative stages: deciding the phenomenon of interest, deciding what is relevant, reading and re-reading the studies, determining how the studies are related, translating the studies into one another, synthesising translations and expressing the synthesis.

During data extraction, second order constructs, defined as, ‘the authors’ interpretations of participants’ accounts often expressed as themes or analytical categories within qualitative studies’, were abstracted from the results and discussion sections of included papers [18]. These related to the study’s aims which were to identify qualitative evidence on the views and experiences of training, barriers and facilitators to its implementation and its perceived impact. Included studies and data extraction tables were then read and re-read. During this process it became apparent that the data related to the perceived impact of training rather than views and experiences of training. After discussion, the data extraction tables were revised to reflect these new themes and the relationships between different papers were considered. For clarity and to demonstrate how the concepts compared with one another, a separate table was created and the data within the original data extraction table were categorised into second and third order constructs to develop a conceptual framework [14, 17]. At this stage first order constructs (quotations) were inserted into the table, to ensure that original data were reflected and to illustrate how third order constructs (interpretations) and the conceptual framework had been developed.

We initially identified 40 emerging themes, which we furnished with first and second order quotes extracted from individual studies. We reviewed these themes and consolidated them into sub-themes, then we applied a line-of-argument synthesis based on the sub-themes [17]. Line-of-argument synthesis involves using inference to construct a picture of the whole (e.g. culture), by using similarities and differences across the component studies. We identified similarities in accounts, but differences in perspectives also emerged from the data, so we applied the line-of-argument synthesis to integrate these findings and derive new insights. We support our findings with direct quotations extracted from the results sections of individual studies where possible. Throughout the synthesis regular meetings were held by the research team to discuss the development of third order constructs.

### Quality appraisal

The appropriateness of assessing quality in qualitative research is a widely debated topic [18–20]. However, if qualitative syntheses are to inform policy and clinical practice, then the quality of the research needs to be determined [21]. Following guidance by the Centre of Reviews and Dissemination which emphasises the importance of a structured approach to quality assessment [22], the Critical Appraisal Skills Programme (CASP) tool [23] was used. CASP consists of a series of ten questions relating to study aims, data collection, data analysis, ethical approval, findings and overall value of the study.

Quality appraisal was undertaken by one researcher and checked by a second. Discrepancies were largely a result of researchers interpreting the CASP tool differently and were resolved through discussion.

## Results

### Search results

**Fig 1:**
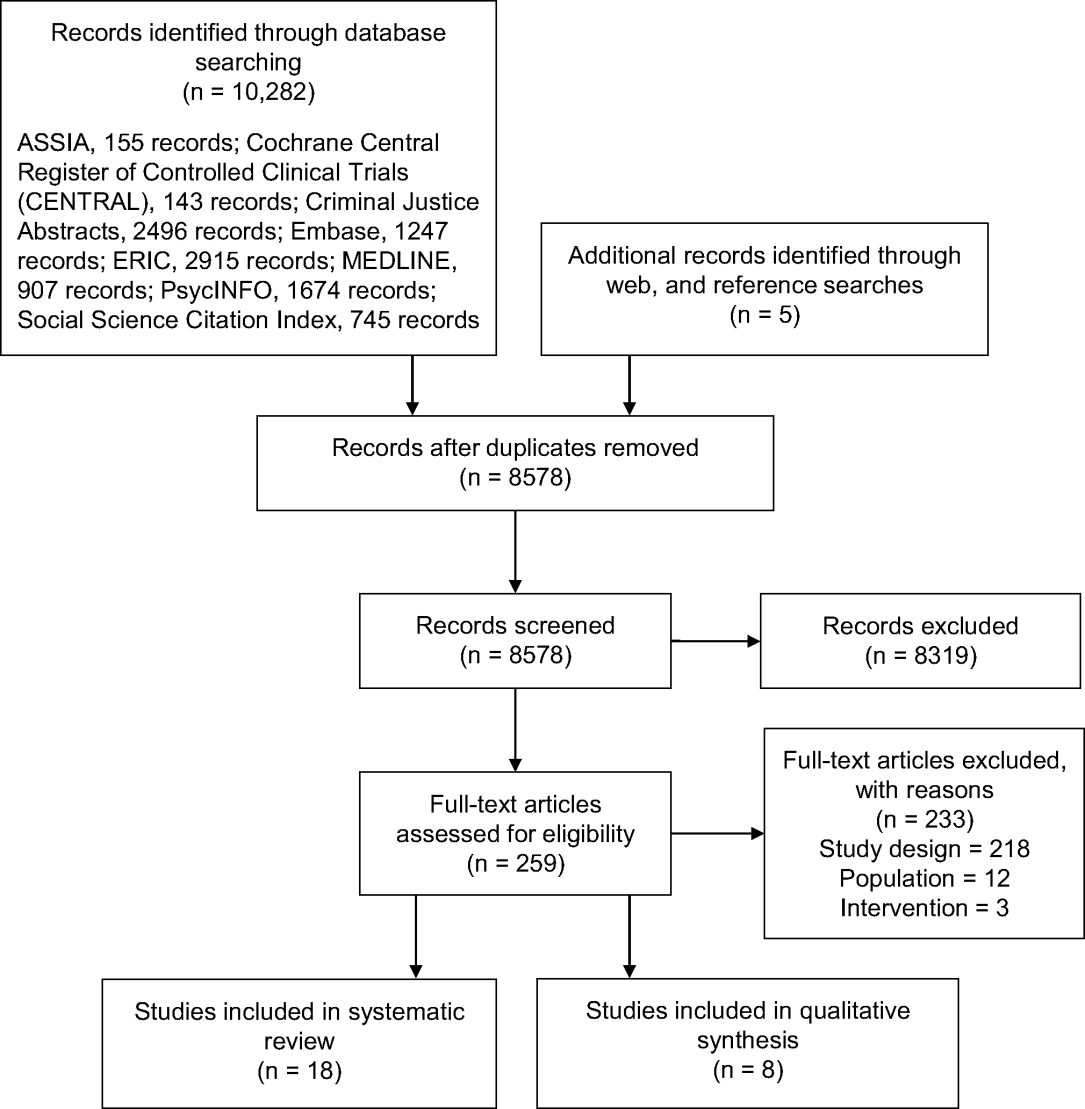
Summary of literature search, adapted from PRISMA [24].

Fig 1 summarises the flow of study selection.

The characteristics of the eight studies that were included in the review are outlined in Table 2. Four of the studies were conducted in the UK [25], three in the US [26] and one in Sweden [27]. Two studies evaluated pre-existing training interventions: CIT [28] [26] and one evaluated Mental Health First Aid [27]. Other studies evaluated specialised training programmes that had not been previously evaluated and which had been designed specifically for prison staff [25], the police [26], care home staff [29] and social workers and carers [27, 30] with one study evaluating inter-professional training for nurses, social welfare, the police and social workers [31].

**Table 2:** Characteristics of included studies.

Reference	Country	Study objectives	Aim of training package	Sample	Method of evaluation	Method of analysis
Svensson, Hansson, Stjernsward (2015)	Sweden	To explore participant’s experiences of Mental Health First Aid Training (MHFAT) by exploring their experiences of the program’s content, suitability in regards to participant’s professional role; format; presentation and impact on knowledge and attitudes.	MHFAT aims to improve mental health literacy in the general population and provide people with the skills necessary to help individuals with mental health issues.	Health professionals, employment agents, social workers, deacon and carers (n = 24)	Focus groups and semi-structured interviews.	Content analysis
Tully & Smith, (2015)	USA	To examine officer perceptions of ‘preparedness’ following Crisis Intervention Team (CIT) training. The underlying factors contributing to officers’ perceptions were also explored.	CIT is a specialised police-based program that aims to enhance officers’ interaction with individuals with mental illness and improve the safety of all those involved in mental health crisis’.	Officers from a single urban police department (n = 8)	Survey, semi-structured interviews	Thematic analysis
Walsh & Freshwater, (2009)^1^	UK	To report on the development and pilot delivery of the ‘Mental Health Awareness for Prison Staff Program’.	To enable officers to identify prisoners at risk of developing and experiencing mental health issues and respond appropriately to the needs of these individuals.	Prison staff (n = 24) from 8 UK prisons and a facilitator	Survey and ‘feedback from course participants and the facilitator’.	Thematic analysis
Anderson, (2014)	USA	To help individuals working within the criminal justice system to develop the tools needed to interact with prisoners with mental health issues, learn the signs and symptoms of mental illness and develop a greater understanding of mental health.	To ensure that staff working within the criminal justice system have the skills and knowledge to work with individuals with mental health issues.	Administrators, individuals that supervise criminal justice personnel and guide police development for the agency. Criminal justice personnel working in custody of a US correctional system. Total participants (n = 83). Qualitative n = 30, quantitative n = 53.	An action research study: Administrator focus group and interviews. Staff focus group and interviews. Staff observations. Survey	Observations, interviews, focus groups: thematic analysis. Survey: descriptive statistics and parametric tests.
Rani & Byrne, (2012)	U.K	To evaluate a newly developed inter-professional training course on dual diagnosis.	To obtain a general understanding of the theoretical and conceptual underpinnings of mental health, substance use disorder and dual diagnosis. To discuss issues surrounding dual diagnosis and evidence based treatment approaches recommended by researchers.	Service providers within Irish mental health and addiction services; nurses, social workers, police and social welfare (n = 20).	Survey, focus group interviews.	Survey: ‘frequencies and percentages’. Focus groups and interviews: thematic analysis.
McGriff et al., (2010)	USA	Identify the knowledge, attitudes and applied skills/experiences in managing mental health crisis situations in a busy airport. To elicit suggestions for improvements to the Crisis Intervention Team (CIT) program for police officers at airports.	To educate police officers to destigmatise mental illness and provide tools for the management of situations involving mental health crisis. CIT aims to provide police officers with the knowledge and skills to enhance their response to individuals with mental illness and safely handle crisis situations. To educate officers about partnerships and collaborations between mental health and the police department as well as other resources to assist them in redirecting individuals with mental illness away from jails and into treatment facilities-where appropriate.	CIT trained police officers at an international airport (n = 9).	Survey and focus groups.	Survey: Descriptive statistics. Focus groups: Content analysis
Macdonald et al., (2011)	U.K	To evaluate the effects of a DVD/manual/coaching skills training programme for carers of people with eating disorders.	Skills based training programme to help carers better manage individuals with eating disorders.	Carers of people with eating disorders (n = 19).	Semi-structured interviews.	Interpretative Phenomenological Analysis.
Gough & Kerlin, (2012)	U.K	To explore the issues around implementation of skills learnt, application of knowledge and maintenance of these new skills/knowledge from the perspective of key stakeholders with managerial responsibility following training on the Mental Capacity Act (MCA).	The specific aims of the MCA training were not stated however, MCA training was introduced by the DoH primarily to aid implementation of the Act.	Managers/deputy managers working in local authority care homes for older people and key stakeholders with responsibility over delivery of training (n = 13).	Focus groups (n = 9), Semi-structured interviews (n = 4).	Grounded theory.

^**1**^ This study has been included. However, it is unclear whether the survey included open or closed questions.

### Quality appraisal outcome

The quality of the papers was variable, and the CASP tool identified methodological weaknesses in all of the studies (Table 3). Common weaknesses were: a lack of rigour in data; unclear descriptions of the data collection methods; failure to consider the relationship between the researcher and participants and failure to consider ethical issues.

**Table 3:** Quality appraisal using the CASP tool.

Source paper	1. Was there a clear statement of the aims of the research? (Yes/No/Can’t tell)	2.Is a qualitative methodology appropriate? (Yes/No/Can’t tell)	3. Was the research design appropriate to address the aims of the research? (Yes/No/Can’t tell)	4. Was the recruitment strategy appropriate to the aims of the research? (Yes/No/Can’t tell)	5. Was the data collected in a way that addressed the research issue? (Yes/No/Can’t tell)	6. Has the relationship between researcher and participants been adequately considered? (Yes/No/Can’t tell)		7. Have ethical issues been taken into consideration? (Yes/No/Can’t tell)		8. Was the data analysis sufficiently rigorous? (Yes/No/Can’t tell)	9. Is there a clear statement of findings? (Yes/No/Can’t tell)	10. How valuable is the research?
Svensson, Hansson, Stjernsward (2015)	Y	Y	Y	Y	Y		N		Y	Y	Y	Provides a valuable insight into the reasons for the positive effect of the training found during the RCT. Facilitators/positives of the training delivery were cited.
Tully & Smith, (2015)	Y	Y	Y	Y	Y		N		Can’t tell	N	Y	Provides recommendations for future CIT or mental health training and evidence of positive impacts of training on knowledge and attitudes of officers towards mental illness.
Walsh & Freshwater, (2009)	Y	Y	Can’t tell	Y	Can’t tell		N		Can’t tell	Can’t tell	Y	Provides information of factors to facilitate training delivery i.e. having skilled facilitators.
Anderson, (2014)	Y	Y	Y	Y	Y		N		Y	Y	Y	Provides a far more comprehensive training protocol than original–all based on requirements of the key staff involved.
Rani & Burne, (2011)	Y	Y	Y	Y	N		Can’t tell		Can’t tell	N	Y	Alludes to key aspects which should be considered when putting together training, in particular the impact of service users & the role they can play in improving understanding.
McGriff et al., (2010)	Y	Y	Y	Y	Y		Can’t tell		Can’t tell	Y	Y	Provides clear benefits of providing training in the resolution of potential situations
Macdonald et al., (2010)	Y	Y	Y	Y	Y		Y		Can’t tell	N	Y	Provides information about how training can be provided to non-trained individuals & how simple coaching can have added value. It also shows how a single intervention is not going to cover all scenarios & the long term nature of mental health means that expectations of the benefits of interventions need to be managed.
Gough & Kerlin, (2012)	Y	Y	Y	Y	Y		Can’t tell		No	Can’t tell	Y	Highlights the importance of contextualising the training & the relevance of it to the people being trained. They also highlight the importance of assessing understanding & practical application once people have attended training. The paper discusses some of the barriers to training including time, money &ability to recognise the relevance of the training to the staff

### Analysis and results

Three key themes emerged from our synthesis, which we discuss:

1. Barriers to training (Table 4)
2. Facilitators to training (Table 4)
3. Perceived impact of training (Table 5)

Tables 4 and 5 outline first- and second-order constructs and third-order synthesised themes.

**Table 4:** Barriers and facilitators to training delivery and implementation.

Third order construct: synthesis of main findings in an explanatory framework	Sub-themes of third order constructs	Second order constructs: interpretations of original findings	First order construct: quotations supporting researchers interpretations^1^
Training Content	***Repetitive***	The program’s structure was viewed as repetitive but valuable for recapping-particularly for those with previous experience of dealing with individuals with mental health problems (Svensson, Hansson, Stjermswald 2015).	
	***Modules and specific content***	Training should be based on needs in the field and presented in a way that allows information to be processed. For example; protocol based training, more detailed information regarding explanations of mental health disorders and the purpose of specific treatments. Participants also suggested that training could be combined with other training to make it more focussed. More information relating to how training corresponds with decreased mental health problems was suggested. A more tailored approach that includes topic specific training for current institutional problems (e.g. drug epidemics) was suggested along with more emphasis on teaching laws, policies and procedures specific for mental health. Staff wanted training to vary, reflecting the different types of inmates they encounter. To facilitate understanding of decision making training should be mental health and not just crisis focussed. Training should also include immediate tactical skills to equip staff in the event that no mental health staff are available-a common barrier to escalation in crisis (Anderson, 2014).	
		Delivery of training was not tailored to the needs of the audience. Mental Capacity Act training should not be treated as standalone training or isolated topic as many of the issues are relevant to all aspects of care. Treating the training as a separate topic was viewed to negatively affect the ability to apply the training. An integrated approach was considered essential to enable staff to make connections between different topics and issues (Gough & Kerlin, 2012).	***“****It doesn’t help to see things in isolation*. *A lot of stuff is thrown at managers*, *such as ‘we’re going to focus on Mental Capacity*, *now dementia*, *then something else’*. *Things are not necessarily joined up so people end up talking very passionately about stroke*, *for e*.*g*. *and are unable to make the connection with Mental Capacity or safeguarding” (Gough & Kerlin*, *2012)*.
Additional resources	***Course manual/workbooks***	High levels of acceptability for the manual which was perceived to provide carers with a flexible, practical and user friendly guide. However, having the time to read the manual was an issue for some. Suggested improving the organisation and delivery of materials (Macdonald et al., 2010).	*“I suppose my main problem was actually finding the time when I could actually watch them and read the book without getting too distracted” (Macdonald et al*., *2010)*.
	***Video/DVD scenarios***	High levels of acceptability for the DVD, which was described as a useful visual aid to the manual but sometimes required more planning for it to be used. Some issues with having the time to watch the DVD were reported. Participants suggested improving the role play with more realistic scenarios or consider other scenarios. For instance, where the carer might want to help with something not necessarily directly related to the sufferer. Some carers described the intervention tools as ‘dull, low’ laborious, tedious’ and felt that the tools had very limited effect because the person they were caring for was still unwell. It was felt by some carers that the scenarios portrayed in the DVD’s were not realistic enough and did not portray the reality some of them faced or that it was just not relevant to them. Practical criticisms of the DVD included its duration, poor quality, carers could not stop and start them, language described as inaccessible and was felt the material was aimed at females and parents (Macdonald et al., 2010).	*“Whereas the DVD is more…you just kind of sit through and watch it and follow it through and sometimes it’s a bit frustrating because erm things*, the role plays don’t necessarily reflect what goes on in your own house” *Macdonald et al., *2010)*.
Training Delivery	***Length***	Participants preferred 4x3 hours with weekly meetings rather than whole days as this was viewed as too intense without room for reflection and information processing. Others preferred a two day approach as it was easier for time (Svensson, Hansson, Stjermswald 2015).	
	***Method***	Participants reported a need to move away from a conventional approach of delivery, as this was considered too abstract in relation to applying knowledge into practice. A targeted approach was seen as important to ensure learning, with a more direct association to the workplace viewed as important. Real life case scenarios were also considered important to provide examples and facilitate the application of learning within the workplace (Gough & Kerlin, 2012).	***“****We have to start looking at more alternative and blended approaches*. *I think we have to stop looking at that old fashioned way of looking at the face to face (training) delivery getting everyone looking into a central point” (Gough & Kerlin*, *2012)*.
		Power points were the least preferred method (Rani & Byrne, 2011).	
	***Trainers/Instructors***	Experienced and knowledgeable instructors described as a prerequisite for the training’s impact and credibility and essential to being able to answer participant’s questions (Svensson, Hansson, Stjermswald 2015)	***“****One mistake was that the ones holding the course didn’t have more experiences of mental ill health than I did*. *They were candid about it*, *but insecure*.*/*..*/Maybe they were not so experienced*, *they couldn’t answer follow-up questions*. *In future courses*, *there should be more experienced instructors*, *both for their own sakes and for ours (Svensson*, *Hansson*, *Stjermswalk*, *2015)*.*”*
		Some participants felt there was a lack of guidance on the coaching procedure and the coach could not provide answers to the situation they were currently managing (Macdonald, et al., 2010).	*“Yeah like of…consistent approach and also not being able to get any dialogue…there were some fairly closed answers P gave me that was basically ‘well go and try this’ and that was it really” (Macdonald et al*., *2010)*.
Staff willingness to engage with training	***Reluctance to change***	Following the course two participants reported that they would not change their behaviour following training-reluctance to admit the need for change was raised as a potential barrier (Walsh & Freshwater, 2009).	
Organisational factors	***Culture***	Participants had limited success in instigating psycho-education groups in their place of work including lack of time, workload, maintaining continuity of the group due to poor attendance. They suggested options for keeping clients engaged (setting up a social group, help clients with physical/social issues, provide creative art materials). (Rani & Byrne, 2011).	***“****It’s one thing reading it in the book and going ‘right OK*, *OK this sounds pretty simple’ and then you might sit down and do it and its gonna take you like two hours to have this conversation because it’s such a tricky one” (Macdonald et al*., *2010)*.
		Carers talked about how their daily life could get in the way of implementing some of the suggestions; so although it sounded simple to implement sometimes the situation was more complex and so it took longer to use. To utilise the intervention effectively carers needed the opportunities to do so but as the sufferer did not live with them made it more difficult (Macdonald et al., 2010).	
		The culture and practice of care homes was considered ‘critical’ to the successful implementation of training, with a perceived gap between those that implement well and those that implement poorly (Gough & Kerlin, 2012).	*“It is patchy*. *What you’ll find is that those homes that do it generally do it well and those who don’t don’t*. *They don’t do anything almost” (Gough & Kerlin*, *2012)*.
	***Managerial and staff buy-in***	A ‘top-down approach’ was considered crucial by some participants, who emphasised the need for managers to buy-into and understand the training (Gough & Kerlin, 2012).	*“I think the training/development of managers Is cruicial and critical and not just around Mental Capacity*. *It’s about the managers being professional in their role and seeing the importance of good practice and good quality care; seeing this as an integral part of their role and promoting that at every turn*. *MCA and DoLS would be part of that” (Gough & Kerlin*, *2012)*.
	***Time and cost***	Time and cost of attending training. For instance, relieving staff to attend training courses is an issue as lose time and money. More problematic for smaller care homes due to smaller numbers of staff and budgets. Mangers needed to consider what would be the most beneficial training for staff to attend. (Gough & Kerlin, 2012).	*“The problem is that I think homes find it difficult to release people for that training (Gough & Kerlin*, *2012)”*.

^1^First order constructs were provided where available.

**Table 5:** Perceived impact of training.

Third order construct: synthesis of main findings in an explanatory framework	Sub-themes of third order constructs	Second order constructs: interpretations of original findings	First order construct: quotations supporting researchers interpretations^1^
Perceptions of mental health	***Understanding***	Participants were divided as to whether they felt the training contributed to a more detailed understanding of mental health (Svensson, Hansson & Stjermsward, 2015).	*“I had an understanding (of mental ill health)*, *but now I see many different types of ill health*, *I read people in a different way” (Svensson*, *Hansson & Stjermsward*, *2015)*.
			*“Why we/…were disappointed*, *it was mainly because we expected that the training would be useful for us*, *that we would receive advice*, *but it seemed to be more for the general public*.*/*..*/As firefighters we’ve already touched upon these subjects in our training/…/*. *We didn’t get much new knowledge targeted at our profession” (Svensson*, *Hansson & Stjermsward*, *2015)*.
		A number of officers felt CIT increased their understanding of mental health (Tully & Smith, 2015).	*“CIT is an asset*, *you are more understanding because of it*. *“(Tully & Smith*, *2015)*.
		Increase in psychiatric knowledge (McGriff et al., 2010).	*“Then with the training*, *it helps you change your approach and gives you an understanding*, *[so that] if someone doesn’t respond to you*, *it’s not like they are resisting you all the time*. *Sometimes they may be off their medication and you try to figure out if that’s their problem versus someone just bring resistive*, *evasive and all that (McGriff et al*., *2010)*. *“*
		Increased knowledge and understanding (Macdonald et al., 2010).	*“Increased awareness*, *knowledge and understanding” (Macdonald et al*., *2015)*.
		Participants reported having improved understanding of mental health problems (Walsh & Freshwater, 2009).	*“I will have a better understanding of mental illness rather than just being ‘a nutter’ (Walsh & Freshwater*, *2009)*.*”*
	***Empathy***	Participants felt the training increased their ‘awareness, understanding and humility’ towards individuals with mental health problems. The majority of participants felt the training promoted reflection about personal courage and responsibility in meeting affected individuals and the importance of seeing the person behind the illness (Svensson, Hansson & Stjermsward, 2015)	*“I had an understanding (of mental ill health)*, *but now I see many different types of ill health*, *I read people in a different way” (Svensson*, *Hansson & Stjermsward*, *2015)*.
		Participants reported a personal transformation from being judgemental to non-judgemental in regard to mental health problems (Walsh & Freshwater, 2009).	*“I will have a better understanding of mental illness rather than just being ‘a nutter’ (Walsh & Freshwater*, *2009)*.*”*
		Participants felt that the training improved officers understanding and compassion towards individuals with mental health problems (Tully & Smith, 2015).	*“CIT has opened my eyes about mental illness and has even made me more patient with my son who has ADHD”*. *“CIT has helped us understand what the mentally ill are going through; it helps you to understand what may be going on in their head” (Tully & Smith*, *2015)*.
		Participants cited a number of attributes such as ‘sensitivity, patience, empathy and compassion’ that underlie the reason for working with individuals through CIT (McGriff et al., 2010).	*“[CIT] gives you that passion to help*, *but I also has to come from within” (McGriff et al*., *2010)*.
		The intervention enabled ‘shared empathy’. Participants also reported how the intervention made them feel ‘less alone’ and that they were not the only people going through these issues (Macdonald et al., 2015).	*“…incredibly important to talk to somebody who had the experience of the issues which I am sure isn’t always the case but in this particular case I found it particularly useful because P started off and told me straight away about her daughter…*.*which I found totally acceptable…*.*Don’t think it was unprofessional or anything like that*. *It was*, *it made it much easier to talk to her really” (Macdonald et al*., *2015)*.
	***Stigma***	Those with previous experience of individuals with mental health problems didn’t feel the training altered their view or approach towards affected individuals, but reinforced how they had handled situations previously. Others felt they were made aware of their prejudice towards individuals with mental health problems, with the training adding new insights and useful advice. The majority of participants felt the program counteracted prejudice and defused the subject of mental health, whilst facilitating a dialogue about mental health problems in professional and private setting (Svensson, Hansson & Stjermsward, 2015).	*“In retrospect*, *I reflected upon things and got an explanation about certain behaviours after the training*. *Simultaneously*, *the prejudice that mentally ill persons are dangerous-I was one of them–I…I*, *it’s not as dangerous anymore*, *after the training” (Svensson*, *Hansson & Stjermsward*, *2015)*.
Response in situations involving mental health	***Management and involvement in situations involving mental health***	Officers felt that time spent on calls increased but the interaction was less problematic. Officers felt CIT improved communication with individuals with mental health problems (Tully & Smith, 2015).	
		Two participants said they would not be changing their practice following training but did not elaborate, others said they would do things differently (Walsh & Freshwater, 2009).	*“Training increases time on a call because we take the time to get more history and spend the time trying to understand the person” (Tully & Smith*, *2015)*.
		Increases to their knowledge of mental health helped to inform and organise how participants dealt with individuals displaying threatening or potentially harmful behaviours. Officers therefore stated how they not only understood the symptoms but recognised the need to adjust their method of interviewing and handling these situations. Participants also stated how their improved understanding helped them to assess situations and make decisions about how to de-escalate situations (McGriff et al., 2010).	*“I guess the most important information that I received when I went through the program is how it [medicine] helps them out*, *how it affects them*, *how their behaviour changes when they don’t take it*, *and how they change when they’re on the medications and when they are not*. *I have a relative*, *she was born with some mental problem*, *which I didn’t understand much until I went to the course and learned what actually they go through and why their behaviour changes and all that kind of stuff*, *and how do you go about dealing with them and interacting with them (McGriff et al*., *2010)*.
		Since the training participants found it easier to ask patients questions about drug use, reported they were more able to recognise symptoms of mental health problems and felt more comfortable challenging the diagnosis made by other agencies (Rani & Byrne, 2011).	
		Some carers reported reduced levels of stress and anxiety. The training the carers received affirmed the behaviour they were already engaging in (Macdonald et al., 2010).	
	***Communication***	Officers felt CIT improved communication with individuals with mental health problems (Tully & smith, 2015).	*“CIT is an asset; you are more understanding because of it*. *CIT teaches valuable communication techniques that may reduce officer injury “(Tully & Smith*, *2015)*.
		Participants reported that since the training they were more comfortable talking about mental health with clients and asking questions about substance abuse with a patient who has mental health problems (Rani & Byrne, 2011).	*“…One of the clients asked me*, *do you think I am mad? …and I said what is mad*, *and we talked about normal*, *mental health and mental illness…I think I was more comfortable talking to him about mental health issues than before” (Rani & Byrne*, *2011)*.
		Participants reported asking more questions, to try and identify issues and get the right information (McGriff et al., 2010).	*“I ask more questions to try and find out what the problem is and see if I can identify it*. *So I just ask more questions and really try to explain to them that I am not there to harm them*. *That I’m trying to help*, *if they let me help them*. *(McGriff et al*., *2010)”*.
		Communication improved because of using the intervention, helping participants to identify ways to communicate more effectively with the sufferer (Macdonald et al., 2010)	*“I think probably*, *you know*, *conversations with her*, *the DVD has helped find the right phrases to use or the right way to approach her*. *(Macdonald et al*., *2010)”*
		Participants viewed the program as a toolbox that led to improved language amongst colleagues The majority of participants felt the program counteracted prejudice and defused the subject of mental health problems, whilst facilitating a dialogue about mental health in professional and private setting (Svensson, Hansson & Stjermsward, 2015).	*“It’s not like I wouldn’t have dared to ask previously*, *I just haven’t thought about it*, *that I should ask*. *I think I can feel that the inquiry (about mental health/suicide thoughts) was appreciated” (Svensson*, *Hansson & Stjermsward*, *2015)*.
		Participants reported feeling more confident talking to individuals about mental health and mental health problems (Rani & Byrne, 2011).	*“…One of the clients asked me*, *do you think I am mad*? …*and I said what is mad*, *and we talked about normal*, *mental health and mental illness…I think I was more comfortable talking to him about mental health issues than before” (Rani & Byrne*, *2011)*.
		Observations supported assumptions during interviews and focus groups that providing mental health training could improve interactions between themselves and prisoners with mental health problems (Anderson, 2014).	
	***Problem solving***	Improved ability to assess the patient’s potential mental health and make decisions to de-escalate situations (McGriff et al., 2010).	*“Once you establish and you know that they have…that this person is not all right or something is going on here*, *so you start asking the questions*, *then you end up talking about medications and all that stuff (McGriff et al*., *2010)”*.
		Since the training participants reported they were more able to recognise symptoms of mental health problems and felt more comfortable challenging the diagnosis made by other agencies (Rani & Byrne, 2011).	*[When speaking of their ability to problem solve pre-training}…”We would have a big problem with Crack at the moment so when a person comes depressed to your programme*, *you don’t know if they are depressed because of coming down from the Crack or if they are genuinely depressed” (Rani & Byrne*, *2011)*.
	***Confidence***	Participants viewed the program as a toolbox that led to increased confidence, an inclination to act to help a person with mental health problems, clarified individual responsibility, (Svensson, Hansson & Stjermsward, 2015)	*“Previously I wouldn’t have wanted to ask or talk about it (suicide thoughts/plans) but now I do” (Svensson*, *Hansson & Stjermsward*, *2015)*.
		Increased confidence in dealing with prisoners suffering from or at risk of mental health problems (Walsh & Freshwater, 2009).	
		Since the training participants found it easier to ask patients questions about drug use, reported they were more able to recognise symptoms of mental health problems and felt more comfortable challenging the diagnosis made by other agencies (Rani & Byrne, 2011).	*“…One of the clients asked me*, *do you think I am mad*? …*and I said what is mad*, *and we talked about normal*, *mental health and mental illness…I think I was more comfortable talking to him about mental health issues than before” (Rani & Byrne*, *2011)*.
		Carers reported increased confidence and self-esteem (Macdonald et al., 2010).	*“It gave me confidence or more confidence and through me*, *my partner and through us…we all got a bit more confidence that we could actually challenge this “(Macdonald*, *et al*., *2015)*.
	***Applying skills learnt during training in practice***	Svensson, Hansson & Stjermsward, 2015includes a number of vignettes describing how participants made practical use of the course to apply their knowledge positively with individuals with mental health problems. Participants felt the program offered new insights and useful advice (e.g. to remain calm and not show fear during crisis situations.	*“I feel a confirmation that I feel right or think right*. *You can need to recapitulate your knowledge with regular intervals*.*/…/*. *You can forget that you need to ask (about mental illness/suicide thoughts or plans)*. *(Svensson*, *Hansson & Stjermsward*, *2015)*.*”*
		All individuals who were observed demonstrated ‘at least a limited use of the mental health intervention strategies learned during mental health training’. Limited use was considered as demonstrating at least 6/10 essential mental health strategies in each observation (Anderson, 2014).	
		The intervention ‘affirmed’ how they had been responding to symptoms and reinforcing their skills. Those who received the intervention referred to the usefulness of the action planning and goal setting; benefit of the personal contact (giving them an opportunity to embed what they had learned in the DVD manual in their understanding); and boosting self-reflection, challenging comfort zones and limiting self-beliefs Macdonald et al (2010).	*“So what I found from the DVDs*, *whilst they were very good at teaching me or confirming some of the skills I’ve already gained” (Macdonald*, *et al*., *2015)*.
Impact	***Impact on potential ‘harm’***	Officers did not feel CIT decreased officer injury, which was attributed to the unpredictability of human behaviour and was not perceived to be solvable by training. Participants felt that the training did reduce civilian injury through rapport building with family and increased officer compassion (Tully & Smith, 2015).	*“CIT does not do much to reduce officer injury because this is something that can never be predicted” (Tully & Smith*, *2015)*.
	***Measuring impact***	Training lacked measurable outcomes-managers were unclear what level of comprehension staff had following training and felt the application to practice also needed to be observed (Gough & Kerlin, 2012).	*“The thing is you go away to your course*, *come back and don’t think about it again for however many weeks*, *and as a manager I cannot gauge where my staff understood (Gough & Kerlin*, *2012)*. *“*
	***Value of training***	Participants said they would recommend the training program for the general public and colleagues form other occupations (Svensson, Hansson & Stjermsward, 2015).	
	***Perceived identification of self***	Having had the CIT training, police officers saw themselves as different to non-CIT officers (Mcgriff et al., 2010).	
		Some participants felt they were made aware of their prejudice towards individuals with mental health problems. The majority of participants felt the program counteracted prejudice and defused the subject of mental health problems, whilst facilitating a dialogue about mental health problems in professional and private settings. Participants felt the training increased their awareness, understanding and humility towards individuals with mental health problems. The majority of participants felt the training promoted reflection about personal courage and responsibility in meeting affected individuals and the importance of seeing the person behind the illness (Svensson, Hansson & Stjermsward, 2015).	*“In retrospect*, *I reflected upon things and got an explanation about certain behaviours after the training*. *Simultaneously*, *the prejudice that mentally ill persons are dangerous-I was one of them–I…I*, *it’s not as dangerous anymore*, *after the training” (Svensson*, *Hansson & Stjermsward*, *2015)*.
		The intervention increased self-reflection challenging comfort zones and limiting self-beliefs. The intervention also prompted a requirement for individuals to change and make changes within their family (Macdonald et al., 2015).	*“…eh it*, *I would say the absolute key thing was around the requirement to change…*.*and it also gave me the additional focus of where changes within the family that needed to change was absolutely the key…key point” (Macdonald et al*., *2015)*.
		Participants reported a personal transformation from being judgemental to non-judgemental in regard to mental health problems (Walsh & Freshwater, 2009)	*“I will have a better understanding of mental illness rather than just being ‘a nutter’(Walsh & Freshwater*, *2009)*.*”*

1 First order constructs were provided where data were available.

### 1. Barriers to training delivery and implementation

There were a number of barriers to training delivery and implementation: training content; training delivery; additional resources; staff willingness to engage with training and organisational factors.

#### 1.1. Training content

A clear emphasis was that the training needed to be tailored to the needs of the trainees, their work context and the people they come into contact with, to ensure its usefulness and future application in practice. There were two key facets to this; firstly that training lacked focus in terms of the requirements of the trainee [32], or that it is treated as a standalone training and so fails to take into account the wider context or other relevant aspects of practice [29]. An integrated approach which linked the training to other issues and/or the wider context was also considered important in enabling trainees to apply the training. Whilst this may mean certain aspects of training were repetitive, it was considered valuable in ensuring that those with prior experience could recap and refresh their skills [27].

*‘It doesn’t help to see things in isolation. A lot of stuff is thrown at managers, such as ‘we’re going to focus on Mental Capacity, now dementia, then something else’. Things are not necessarily joined up so people end up talking very passionately about stroke, for e.g. and are unable to make the connection with Mental Capacity or safeguarding’* (Gough & Kerlin, 2012).

#### 1.2. Training delivery

There were issues around the delivery of training including: length of training; method of delivery and course instructors. Different trainees expressed different preferences around the length of training. For some, whole days dedicated to the training were regarded as ‘too intense’, and gave insufficient scope to process the information and reflect on the training; whilst others preferred condensed delivery over two days [27]. Conventional methods of training delivery were also regarded as too abstract to encourage trainees to apply the knowledge in practice [29]. To address this, targeted approaches which made a direct association to the workplace or practice context were deemed important, as were real-life scenarios, which were seen to be better for facilitating implementation of the training within the workplace [29]. Course instructors in some instances were perceived to not provide sufficient guidance and to not be able to provide answers to the situations being managed by trainees [30]. Experienced and knowledgeable instructors were regarded as crucial for ensuring the credibility of training and its impact [27].

***‘**One mistake was that the ones holding the course didn’t have more experiences of mental ill health than I did. They were candid about it, but insecure …maybe they were not so experienced, they couldn’t answer follow-up questions. In future courses, there should be more experienced instructors, both for their own sakes and for ours.’* (Svensson, Hansson, Stjermswalk, 2015).

#### 1.3. Additional resources

The time required for trainees to familiarise themselves with additional course materials was considered a barrier. The organisation and delivery of such material was thought to require improvement [30]. Issues around use of DVDs included: duration; poor quality; use of inaccessible language; not being specifically targeted at the trainees; and being difficult to use [30]. Some of the content of the additional resources, such as role plays and the use of scenarios were not considered to be realistic enough and did not reflect the reality of the experiences faced by the trainees, or in some cases were regarded as irrelevant or ‘frustrating’[30].

*‘I suppose my main problem was actually finding the time when I could actually watch them and read the book without getting too distracted.’* (Macdonald et al., 2010).

#### 1.4. Organisational factors

Factors such as time and cost; organisational culture; and ‘buy-in’ from staff and managers were identified as additional barriers to attending the training and implementing training in practice. For some organisations, the time and costs associated with staff attending training was an issue, particularly for those with fewer numbers of staff and smaller budgets [29].

*‘The problem is that I think homes find it difficult to release people for that training’* (Gough & Kerlin, 2012)

Employers’ competing priorities impacted on attendance at training events and meant that employees were not automatically permitted to attend training [29]. There was a perceived gap between organisations that implement training well and those that implement it poorly [29]. Poor implementation was attributed to lack of time, workload, caring responsibilities or not recognising the need for change [25, 29].

### 2. Facilitators to training delivery and implementation

There were a range of factors that were thought to facilitate the delivery and implementation of the training. These included: the training content and delivery; staff willingness to engage with training; and organisational factors.

#### 2.1. Training content

For the content of the training, the following were highlighted as facilitating factors: modules and specific content; additional resources; and use of video scenarios.

The involvement of key stakeholders such as service users and members of the relevant staff group in the development and delivery of the training was perceived to be a key facilitator in promoting acceptance. Gaining insights into the perspective of people with mental health problems was thought to be useful in making the training more ‘real’, as well as promoting the idea of working in partnership with mental health service users [31]. Likewise, the involvement of members of staff in developing the content of training, for example through action research, was appreciated by staff, and helped to prevent feelings of intrusion and promoted teamwork and acceptance [32].

‘*Seeing the programme from the patients’ point of view, “It was an eye-opener”‘. (Rani & Byrne, 2011).*

Generally, the content of the training was thought to be most suitable when it was tailored to the participants’ institution and the common problems faced by participants in their everyday work, such as increases in drugs—‘drug epidemics’. This required training to be varied and adaptable. There was a view that training should be based on needs in the field, adopt an integrated approach to allow participants to make connections between different topics and issues, and be presented in a way that allows information to be understood and applied. For example, more detailed information regarding explanations of mental health disorders and the purpose of specific treatments. Due to prior limited knowledge, training which contained detailed information and explanations around mental health disorders, and which also provides an overview of the purposes of specific treatments was thought to be helpful. It was suggested that training should be mental health and not just crisis focussed and should focus on laws, policies and procedures specific to mental health. The need for immediate tactical skills to enable participants to de-escalate situations in the event of mental health staff not being available was also identified [32]. Other content that was deemed helpful by participants included community orientated content and information relating to local mental health resources [25, 28, 29, 31].

*‘I do not feel I am aware of mental health resources available to us as officers, I would like to have more information.’* (Tully & Smith, 2015)

Additional resources were considered crucial for enhancing training and implementing it in practice. Resources included: course manuals, workbooks, checklists, crib-sheets, DVDs, videos and e-learning and were perceived to provide flexible, practical, useful and acceptable additions to the training content [25, 27, 29–31]. Course manuals were regarded as educational and valuable aide-memoirs, and could be useful prior to the training to enable early self-directed learning; or after the training to enable implementation of the training. Additional material such as checklists supplied during the training could be made available in the workplace and used to support and provide a rationale for decisions or specific courses of action [29].

***‘**The manual can be used as a reference book, if there’s anything one reflects upon. It’s educational and easy to use’.* (Svensson, Hansson, Stjermswald 2015)

Videos were also considered facilitators to training. For instance, filmed video clips featuring the experiences of people with mental health problems were described as helpful in allowing participants to identify with affected individuals [27]. The use of real-life scenarios in videos was also suggested to facilitate the implementation of training in practice. Whilst role plays created some apprehension, participants valued them, especially when they were video recorded and could be reflected on [31].

#### 2.2. Training delivery

Key facilitators for successful delivery of training were the course trainers (also known as instructors or facilitators), method, length and frequency of training.

Trainers experience, skills and knowledge were considered important pre-requisites to facilitating successful training delivery and ensuring its impact [25, 27, 31, 32]. Trainer’s knowledge of the context, culture and terminology of participants’ workplace was thought to be particularly important, alongside an ability to answer participants’ questions and provide specific contextual examples and guidelines:

***‘****He (the instructor) was very good; he gave me guidelines*, *so I knew how I should work*. *I could call the psychiatric services in that case*.*’* (Svensson, Hansson, Stjermswald 2015)

Participants had clear preferences around the frequency and length of training, though views varied. Some preferred training to be delivered in bite-sized segments, spread over a longer period, to enable processing of information and to allow them to manage other priorities [27, 29]; whilst others found training that was condensed over fewer days easier to manage [27]. In terms of frequency, the need to update training through further refresher courses was deemed important [26, 28]. Topics to be covered by refresher courses included: psychiatric disorders; assessment skills, research updates; local community resources for people with mental illness; and enhancing community partnerships between police and mental health providers.

*‘I wish we had more updated training*, *as time goes on I feel the training fade’*. (Tully & Smith, 2015)

The active use of a range of teaching methods that could be adapted to the needs of participants was highlighted [25, 29, 31]. In particular, targeting teaching to the real-life experiences of participants was seen as critical to learning. Teaching methods that moved away from conventional teaching—which was considered too abstract to be applied in practice–towards alternative and blended teaching that included the use of group discussions, role plays, and online-based training combined with in-person teaching and group work were deemed more facilitative. Additionally, real-life scenarios were seen to facilitate implementation of the training within the workplace [29].

***‘****We have to start looking at more alternative and blended approaches*. *I think we have to stop looking at that old fashioned way of looking at the face to face (training) delivery getting everyone looking into a central point’* (Gough & Kerlin, 2012).

#### 2.3 Staff willingness to engage with training

Staff willingness to engage with training may facilitate its implementation in practice and may be influenced by their desire for and understanding of the purpose of training [31, 32]. An important facilitator to training attendance was staff recognising the need to improve their own practice in managing people with mental health problems through developing new skills [31]. Staff understanding of the reasons behind the training was important in facilitating legitimisation of changed practice [32].

#### 2.4. Organisational factors

Organisational factors such as culture, incentives for training, the training environment, time and cost and organisational ‘buy-in’ could help facilitate training and its implementation in practice.

Managerial and staff ‘buy in’ to training, alongside a ‘top-down’ approach where managers promoted training as a core part of employees’ role was deemed important to ensure it was prioritised. Making training mandatory and offering incentives such as increased annual leave and alternative work rotas were suggested to promote engagement [26, 28].

*‘I think the training/development of managers is crucial and critical and not just around Mental Capacity*. *It’s about the managers being professional in their role and seeing the importance of good practice and good quality care; seeing this as an integral part of their role and promoting that at every turn*. *MCA [Mental Capacity Act] and DoLS [Department of Liberty Schemes] would be part of that’* (Gough & Kerlin, 2012).

When individuals did attend training, the culture and practice of the workplace was considered ‘critical’ to the successful implementation of training [29]. Additionally, the training environment was thought to be most facilitative when it was located in-house in the participants’ workplace, and had a relaxed and safe atmosphere which enabled self-disclosure [25, 29].

*‘My feeling is that if you can associate it with the workplace rather than being completely out of the situation it makes you think about your work environment as well’* (Gough & Kerlin, 2012).

### 3. Perceived impact of the training

The perceived impact of the training focused on: perceptions of mental health; response in situations involving mental health; and impact of training on trainees.

#### 3.1 Perceptions of mental health

There were a range of issues around the perception of mental health, such as understanding, empathy and stigma.

Four studies reported that participants generally described an increased knowledge and understanding of mental health [25, 26, 28, 30]. However, in one study participants were divided about whether they felt the training led to increased understanding of mental health [27].

*“Why we…were disappointed, it was mainly because we expected that the training would be useful for us, that we would receive advice, but it seemed to be more for the general public …As firefighters we’ve already touched upon these subjects in our training…. We didn’t get much new knowledge targeted at our profession”* (Svensson, Hansson & Stjermsward, 2015).

Five studies reported that the training increased empathy in trainees [25–28, 30]. There were various elements to this sense of empathy that included: increased awareness, compassion, humility, sensitivity and patience. This could be described as involving a transformation from participants seeing themselves from being judgemental to non-judgemental about people with mental health problems.

*‘I will have a better understanding of mental illness rather than just being a nutter’* (Walsh and Freshwater, 2009).

Most participants in this study described feeling that the training challenged prejudice against people with mental health problems and allayed the tensions around the topic of mental health. Participants often described how the training promoted the importance of seeing the person behind the mental illness, alongside reflection about personal courage and responsibility in working with people with mental health problems. For others, the training enabled a sense of ‘shared empathy’, where they felt less alone in realising that other people also experienced similar issues. One study explored the impact of the training on stigma [27].

*‘I found it particularly useful because P started off and told me straight away about her daughter…which I found totally acceptable…Don’t think it was unprofessional or anything like that. It was, it made it much easier to talk to her really’.* (Macdonald et al., 2015).

#### 3.2 Response in situations involving mental health

Five studies described how the training impacted on how trainees dealt with situations involving people with mental health problems [25, 26, 28, 31]. Two participants in one study reported that they would not change their practice following training and did not elaborate further [25]. However, the majority of participants thought that the training had a positive impact on how they managed interactions with people with mental health problems. For some, improved communication skills meant that the interaction with people with mental health problems became less problematic. For instance, following the training some participants reported being better able to recognise the symptoms of mental health problems and so found it easier to assess situations, adjust their method of handling such situations and make decisions that were more likely to de-escalate a situation. For carers attending the training, a positive impact included feeling affirmed in their caring behaviour and in some cases reduced their levels of anxiety and depression. Some police officers noted that time spent in dealing with people with mental health problems increased following training.

*‘Training increases time on a call because we take the time to get more history and spend the time trying to understand the person*.*’* (Tully & Smith, 2015).

Six studies reported improved communication skills as a key impact of the training course [26–28, 30–32]. A number of studies reported that participants experienced increased confidence following the training [25, 27, 30, 31]. There were various aspects to this, including increased confidence in asking questions about patients’ mental health, as well as dealing with people with mental health problems in general. This increase in confidence occurred in parallel with a greater inclination to help individuals with mental health problems, a greater awareness by participants of their individual responsibility and increased self-esteem. More effective communication skills were considered a valuable asset that could lead to reduction in officer injuries, as well as something that the person with mental health problems appreciated.

*‘I ask more questions to try and find out what the problem is and see if I can identify it*. *So I just ask more questions and really try to explain to them that I am not there to harm them*. *That I’m trying to help*, *if they let me help them’*. *(*McGriff et al., 2010).

Three studies reported how participants applied the skills learned during the training in their working practice [27, 30, 32]. In general, participants were reported to make at least some use of the strategies learned during the mental health training. For a number of participants, the training served to reinforce existing skills and provide affirmation of their previous responses to people with mental health problems. There were other specific, practical skills that participants reported using and valued in their working practices, such as asking people with mental health problems specifically about suicidal thoughts or plans.

*‘I feel a confirmation that I feel right or think right*. *You can need to recapitulate your knowledge with regular intervals*.*/…/*. *You can forget that you need to ask (about mental illness/suicide thoughts or plans)’* (Svensson, Hansson & Stjermsward, 2015).

Another impact of the training, reported in two papers was participants’ improved ability to recognise symptoms of mental health problems, enhanced skills in assessing the situation and better make decisions to de-escalate potentially volatile situations [26, 31]. Other elements of the training that were perceived to be particularly useful included advice on how to remain calm, not showing fear during a crisis situation, action planning and goal setting.

*‘Once you establish and you know that they have…that this person is not all right or something is going on here*, *so you start asking the questions*, *then you end up talking about medications and all that stuff*.*’* (McGriff et al., 2010).

#### 3.3. Impact of training on trainees

A number of studies positively reported on the impact of the training on trainees perceptions of themselves [26, 27, 30]. The training was thought to encourage self-reflection and increase awareness of their own prejudice towards people with mental health problems, with these perceptions challenged and counteracted during the course of the training. This led to participants changing their view of people with mental health problems from being judgemental to non-judgemental, as well as increasing their understanding, awareness and humility towards people with mental health problems. Following the training, some participants saw themselves as different from those who had not undergone the training.

*‘In retrospect*, *I reflected upon things and got an explanation about certain behaviours after the training*. *Simultaneously*, *the prejudice that mentally ill persons are dangerous-I was one of them–I…I*, *it’s not as dangerous anymore*, *after the training*.*’* (Svensson, Hansson & Stjermsward, 2015).

The lack of perceived impact of the training on reducing police officer injury or physical harm was discussed in one study [28]. This lack of perceived impact was thought to be a consequence of human behaviour being difficult to predict and not readily resolvable through training. However, the training was reported to have a positive impact on reducing injuries of people with mental health problems, thought to be a result of improved dialogue with family and increased compassion in officers. Reflecting the largely positive perceptions of the training and its impact, one study reported that trainees valued the training programme and would recommend it to both colleagues from other disciplines and members of the general public [27].

*‘CIT does not do much to reduce officer injury because this is something that can never be predicted’* (Tully & Smith, 2015).

Although, the majority of studies reported a range of impacts of the training, one study reported that the training lacked measurable outcomes and as a result managers were unclear what levels of understanding participants achieved post-training. Managers therefore reported that the application of the training to working practice needed to be observed [29].

*‘The thing is you go away to your course*, *come back and don’t think about it again for however many weeks*, *and as a manager I cannot gauge where my staff understood’*. (Gough & Kerlin, 2012)

## Discussion

### Summary of key findings

We reviewed the qualitative evidence on the views and experiences of non-mental health professionals, receiving mental health training and the barriers and facilitators to training delivery and implementation. There were eight included studies that used focus groups, interviews observations and surveys. The studies were undertaken in the UK, US and Sweden and the training programmes were targeted at a range of occupational groups including the police. The barriers and facilitators to training delivery and implementation identified largely relate to: training content; training delivery; training method and organisational factors. Staff willingness to engage with training and the provision of additional resources such as the time required for trainees to familiarise themselves with additional training materials were also identified as facilitators and barriers to training respectively. The review originally sought to identify evidence on views and experiences of non-mental health professionals receiving training. However, this was not reported in included studies and instead data reflected the perceived impact of training. This included impact on trainees’ perceptions of mental health as well as impact on response in situations involving people with mental health problems. Exploring the perceived impact of training not only provides insight into the potential effect training may have on participants, but may also be a useful method for identifying outcomes of training that could be assessed in future evaluations. For example, the impact of training on stigma and empathy. The quality of the included literature was variable, with methodological weaknesses and issues with reporting commonly identified.

### Comparison with existing literature

This meta-synthesis focusing on the qualitative evidence is designed to complement and provide additional insights to a wider systematic review of the quantitative evidence on the effectiveness of mental health training programmes for non-mental health specialists being undertaken by our team[8]. The qualitative evidence corresponds with a number of studies that suggest that training interventions that include dramatisations and role play are beneficial for learning [33–38]. Our review also highlights that training which is delivered using a range of delivery methods, a mixture of resources and interactive elements is valued. This is consistent with a review of 58 RCTs, which concluded that for optimum learning training should be delivered using a range of delivery methods, in groups of less than 40, in applied settings over 20 hours on multiple occasions [39].

The importance of skilled trainers for ensuring successful training delivery and impact was also emphasised in our review, with trainer’s knowledge of the context, culture and terminology of participant’s workplace deemed important. This corresponds with existing quantitative evidence of training for police officers, which nearly all used police trainers alongside mental health professionals to facilitate understanding of different organisational cultures [8].

Our meta-synthesis adds to the very sparse literature on the impact of mental health training interventions and we are not aware of any other systematic reviews of qualitative evidence on the same topic. A number of studies in our review reported that training may have a positive impact on trainees through improving their knowledge, empathy, and stigma towards people with mental health problems and their ability to recognise signs and symptoms. Our review of the quantitative evidence concluded that there may be some short term change in behaviour for the trainees, but calls for more high quality RCTs to evaluate the impact of training programmes for non-mental health professionals coming into contact with people with mental health issues. The systematic review also identified the difficulties in evaluating the impact of mental health training and recommended that studies have a longer length of follow up and encouraged the development of a set of core outcome measures.

### Strengths and limitations

By undertaking a meta-synthesis of the qualitative evidence, an in-depth understanding of participants’ perceptions of the barriers, facilitators and impact of training has been achieved, which may not have been possible through considering only the quantitative evidence. For example the qualitative studies provide insight into how the training was perceived to reduce trainees’ stigma towards people with mental health problems.

A limitation of the review is the quality of the included studies, which in turn may have limited the strength of the recommendations and conclusions drawn. A further limitation of the study is that quality appraisal was undertaken by one researcher and verified by another, rather than by two researchers independently. Whilst our searches were systematic and comprehensive, there is a possibility that some relevant studies may not have been found, or excluded as English language and OECD countries only restrictions were applied. Additionally, the richness of the data collected by the included studies may have been affected, as although the review aimed to identify barriers and facilitators to training, this was not necessarily an aim of the individual studies. However, given the range of barriers and facilitators identified, this is unlikely to have affected the interpretation or reporting of the study’s findings.

### Recommendations

Based on the study’s findings a number of suggestions for organisations to consider when providing mental health training were made (Table 6).

**Table 6:** Recommendations for designing, implementing and evaluating training.

Recommendations	
*Training delivery*	
Trainers	To build trust and to provide specific, practical advice, training could be delivered by skilled individuals (e.g. mental health specialists) with a background/experience in the area of interest. Service users or relevant patient groups could also be involved in training delivery where possible. For organisations where training is delivered in house, external experts and a collaborative approach are particularly encouraged.
Methods of delivery	Adopting different delivery methods and using interactive elements and a mixture of resources was considered useful. Interactive elements and a mixture of resources were considered useful. Skill based-learning to allow practice of skills was also valued. To facilitate this, scenarios and role-plays are suggested and may provide staff with the opportunity, and a safe environment to test what they have learnt. If resources allow, actors and/or service users could be used for role play.
Regular, updated training	Refresher training to update skills was considered important and allows staff to share any new resources or skills since previous training. To avoid perceptions that training is repetitive, it may prove useful to inform staff of the relevance and purpose of refresher courses and to use a range of examples and scenarios.
*Additional resources*	
Resources	‘Take-away’ resources such as course booklets were considered useful for facilitating learning. Training could also highlight useful resources, guidelines and checklists to encourage wider and continued learning.
*Organisational issues*	
Protected time and managerial support for training	Staff protected time to attend training and to undertake self-directed learning if needed was identified as important. This could be implemented by making it clear that protected time is available and specifying that time is allocated to attend the course and complete self-directed learning. The studies included in our review also discussed the potential resource implications associated with providing the time for individuals to undertake training and for providing external trainers, actors for role plays, videos and for conducting evaluations.
Promoting engagement and willingness to attend training	It may prove useful to provide a clear rationale for training and to ask participants their reasons for attending training, to facilitate staff buy-in and engagement.
Reviewing training	This review identified that training may affect individuals’ perceptions of mental health, which may not be detected through quantitatively evaluating training effectiveness. Efforts to determine what has been learnt following training are therefore recommended. Staff and managers may also find it useful to work collaboratively to establish lessons learned and how to apply these lessons in practice following training.

### Implications

The findings of our review demonstrate that following mental health training, individuals’ response to situations involving mental health and their perceptions and ability to recognise mental health problems may change. Evaluations of training should include a qualitative component to ensure that these impacts can be measured. However, given the poor quality of the studies included in this review, it is important that future qualitative studies follow relevant guidance for undertaking and reporting standards [40].

Including a qualitative component within training evaluations is also important to provide insight into how best to assess and interpret the quantitative impacts of training. For example, our review suggests that training may increase the time individuals spend managing or responding to situations involving individuals with mental health problems. If taken in isolation, this could be perceived by organisations, staff and service users as a negative effect of training, when in reality this increase could be due to staff taking longer to deal with situations because of improved communication skills or resource constraints (e.g. insufficient staffing levels). Qualitative components within training evaluations can be implemented using the ‘process evaluation’ framework proposed by the UK Medical Research Council [41], which can help to provide a more detailed understanding of implementation, mechanisms and context issues and inform both policy and practice.

## Supporting information

S1 Checklist PRISMA 2009 checklist. (DOC)

S1 Table ENTREQ statement. (DOCX)

S1 Text Search strategies. (DOCX)

## Abbreviations

CASP: Critical Appraisal Skills Programme
CIT: Crisis Intervention Team
CJA: Criminal Justice Abstracts
CRD: Centre for Reviews and Dissemination
ISRCTN: : International Standard Randomised Controlled Trials Number
MESH: Medical Subject Headings
MHFAT: Mental Health First Aid Training
NHS: National Health Service
OECD: Organisation for Economic Co-operation and Development
RCT: Randomised Controlled Trial
UK: United Kingdom
US: United States
