# Supplementary material for: Implementing mental health training programmes for non-mental health trained professionals: A qualitative synthesis
Source: PLoS One. 2018 Jun 25;13(6):e0199746. doi: 10.1371/journal.pone.0199746 (PMC6016927; doi:10.1371/journal.pone.0199746)
Supplement: S1 Table — (DOCX) [file pone.0199746.s002.docx]

S1 table Enhancing transparency in reporting the synthesis of qualitative research: the ENTREQ statement ^[[1]](#footnote-1)^

| **Number** | **Item** | **Guide and description** | **Response (page no. in manuscript)** |
| --- | --- | --- | --- |
| **1** | Aim | State the research question the synthesis addresses. | To identify and explore qualitative evidence on the barriers, facilitators and perceived impact of mental health training programmes for non-mental health professionals. abstract, p.2; background, p.5 |
| **2** | Synthesis methodology | Identify the synthesis methodology or theoretical framework which underpins the synthesis, and describe the rationale for choice of methodology | Meta-synthesis, Meta-ethnographic approach as outlined by Noblit and Hare was used for the synthesis of included studies Abstract p. 2 Methods, literature synthesis p.6 |
| **3** | Approach to searching | Indicate whether the search was pre-planned | Pre-planned systematic search undertaken by an information specialist. Abstract, p. 2, Methods, searching and identifying relevant studies p.5 Search strategy appendix A, |
| **4** | Inclusion criteria | Specify the inclusion/exclusion criteria | See Methods, Inclusion and exclusion criteria and table 1 p. 5 |
| **5** | Data sources | Describe the information sources used and when the searches conducted; provide the rationale for using the data sources | See Methods “Searching and identifying relevant studies,” p.5. Criminal Justice Abstracts (CJA); MEDLINE; Embase; PsycINFO; ASSIA; CENTRAL; SSCI; ERIC; Campbell Library; Social Care Online and EPOC.  Websites of major health charities were also searched and manual searches of the reference lists of included studies were undertaken. |
| **6** | Electronic Search strategy | Describe the literature search. | The search strategy is included in appendix A and was conducted by an information specialist. |
| **7** | Study screening methods | Describe the process of study screening and sifting. | See Methods, Data extraction, p.5. titles and abstracts and then full papers were independently screened by two reviewers. Discrepancies were resolved through discussion. |
| **8** | Study characteristics | Present the characteristics of the included studies | See Results, search results, p. 7 and table 2. |
| **9** | Study selection results | Identify the number of studies screened and provide reasons for study exclusion | Figure 1 shows the flow of study selection p.7. |
| **10** | Rationale for appraisal | Describe the rationale and approach used to appraise the included studies or selected findings | See Methods ‘quality appraisal p.7)v table 3, page 8 and Results ‘quality appraisal outcome’ p.8 |
| **11** | Appraisal items | State the tools, frameworks and criteria used to appraise the studies or selected findings | The Critical Appraisal Skills Programme (CASP) tool was used for quality appraisal of all included studies Abstract, p.2. Methods, p.7 Results, p. 8 |
| **12** | Appraisal process | Indicate whether the appraisal was conducted independently by more than one reviewer and if consensus was required. | Appraisal was undertaken by one researcher and checked by a second. Discrepancies were resolved through discussion. See Methods ‘Quality Appraisal’ p.7 |
| **13** | Appraisal results | Present results of the quality assessment and indicate which articles, if any, were weighted/excluded based on the assessment and give the rationale. | No articles were excluded on the basis of quality assessment alone. The quality appraisal is discussed in the results ‘quality appraisal outcome’ p.8. |
| **14** | Data extraction | Indicate which sections of the primary studies were analysed and how were the data extracted from the primary studies? | See Methods ‘Data extraction p. 5-6. Full manuscripts were read. Data were extracted into a data extraction form and included information relating to: country, setting, participants, study aims, training intervention, method of evaluation and methodology, views and experiences of training and barriers and facilitators to implementation.  Data were independently extracted by one reviewer and checked by another. |
| **15** | Software | State the computer software used, if any. | Excel, Word and EndNote |
| **16** | Number of reviewers | Identify who was involved in coding and analysis. | AS, AH-M, NM |
| **17** | Coding | Describe the process for coding of data | See p.6 methods ‘literature synthesis’. |
| **18** | Study comparison | Describe how were comparisons made within and across studies | See tables 4 and 5. Data were extracted into tables that included first, second and third order constructs that allowed comparison within and across studies. |
| **19** | Derivation of themes | Explain whether the process of deriving the themes or constructs was inductive or deductive. | See Methods: p. 6 ‘literature synthesis’ and results literature synthesis analysis and results p. 8  Largely deductive as we extracted information relating to: views and experiences of training and barriers and facilitators to its implementation. However, data reflected ‘perceived impact of training’ rather than views and experiences of training. |
| **20** | Quotations | Provide quotations from the primary studies to illustrate themes/constructs, and identify whether the quotations were participant quotations of the author’s interpretation. | Tables 4 and 5 include quotations or first order constructs to demonstrate how the original data is reflected in our own interpretations and to illustrate how the conceptual framework has been developed. We also support our findings with direct quotations extracted from the results sections of individual studies as much as possible during the reporting of the results. |
| **21** | Synthesis output | Present rich, compelling and useful results that go beyond a summary of the primary studies. | Tables 4 and 5 summarise the study’s findings as a conceptual framework. |

1. Adapted from Tong et al., *BMC Medical Research Methodology, 2012,* 12: 181 Appendix B [↑](#footnote-ref-1)
